# Circular RNA in cardiovascular disease: Expression, mechanisms and clinical prospects

**DOI:** 10.1111/jcmm.16203

**Published:** 2020-12-22

**Authors:** Ying Tang, Jinghui Bao, Jiahui Hu, Leiling Liu, Dan‐Yan Xu

**Affiliations:** ^1^ Department of Internal Cardiovascular Medicine The Second Xiangya Hospital Central South University Changsha China

**Keywords:** biogenesis, cardiovascular disease, circRNA, miRNA, RNA‐binding protein

## Abstract

Circular RNAs (circRNAs) are a group of covalently closed, endogenous, non‐coding RNAs, which exist widely in human tissues including the heart. Increasing evidence has shown that cardiac circRNAs play crucial regulatory roles in cardiovascular diseases (CVDs). In this review, we aimed to provide a systemic understanding of circRNAs with a special emphasis on the cardiovascular system. We have summarized the current research on the classification, biogenesis and properties of circRNAs as well as their participation in the pathogenesis of CVDs. CircRNAs are conserved, stable and have specific spatiotemporal expression; thus, they have been accepted as a potential diagnostic marker or an incremental prognostic biomarker for CVDs.

## 1 INTRODUCTION

Despite the success of disease prevention, diagnostic markers and medical advances in treatment, cardiovascular diseases (CVDs) remain the leading cause of morbidity and mortality worldwide.^1^ Thus, pathogenesis mechanisms at the molecular level, potential biomarkers and new therapeutic targets for treating CVDs are urgently needed.

A potential new biomarker and therapeutic target for CVDs are circular RNAs (circRNAs), which are a special class of non‐coding RNAs that were discovered in 1976 by Sanger HL.^2^ CircRNAs are important regulatory elements at both transcriptional and posttranscriptional levels via the circRNA‐miRNA‐mRNA axis and because of their interaction with RNA binding proteins (RBPs). Emerging evidence indicates that circRNAs might exert crucial effects on a number of CVDs including atherosclerosis, coronary heart disease, myocardial infarction, cardiac hypertrophy, heart failure (HF), dilated cardiomyopathy and arrhythmia.^3^, ^4^, ^5^, ^6^

In this review, we offered an overview of the current roles of circRNAs in the cardiovascular system. First, we described the biogenesis, classification and biological characteristics of circRNAs. Next, we summarized the expression of circRNAs in the cardiovascular system, and the molecular mechanisms of circRNAs in the occurrence and progression of CVDs. Finally, we outlined the clinical use of circRNAs as potential biomarkers and therapeutic targets.

## 2 BIOGENESIS, CLASSIFICATION AND CHARACTERISTICS OF CIRCRNAS

CircRNAs are recognized as covalently closed continuous single‐stranded RNA loops that exist in the eukaryotic transcriptome.^7^ In general, circRNAs are stable, conserved between species and have specific spatiotemporal expression.^8^ Unlike linear RNAs that are terminated with 5ʹ caps and 3ʹ tails, circRNAs form loop structures with the 5ʹ and 3ʹ ends covalently closed.^7^ These loops form because of a back‐splicing event differing from the canonical splicing pathway. CircRNAs are classified into four categories based upon the synthesis mechanism: exonic circRNAs (ecircRNAs), circular intronic RNAs (ciRNAs), exon‐intron circRNAs (EIcirRNAs) and intergenic circRNAs.

### 2.1 Biogenesis of circRNAs

CircRNAs are generated through a unique mechanism known as back‐splicing. Recent research has shown that back‐splicing is catalysed by the canonical spliceosomal machinery and modulated by both RBPs and intronic complementary sequences (ICSs).^8^, ^9^ However, unlike canonical splicing, the biogenesis of a circRNA requires that a donor splice site of an exon is not connected to an acceptor splice site of a downstream exon, as observed in linear splicing, but instead to an upstream acceptor site.^10^ As a result of the back‐spliced junction event that forms a unique exon–exon junction not present in the linear transcript, the order of exons in sequencing reads is changed. Moreover, circRNA can contain any number of exons, from none, to a single exon or multiple exons.^11^ Three models of circRNA biogenesis through back‐splicing have been accepted: intron‐pairing‐driven circularization, RBP‐driven circularization and lariat‐driven circularization. (Supplementary file 1).

### 2.2 Classification of circRNAs

The majority of circRNAs are ecircRNAs, which lack intronic sequence in their reads. ciRNAs are formed without exon‐skipping events; therefore, they contain only intron sequences. EIcirRNAs contain both intronic and exonic sequences.^12^ In general, ecircRNAs, ciRNAs and EIcirRNAs are all intragenic circRNA arising from the sequences within the parental gene locus. Different from the location of intragenic circRNAs in the genome, intergenic circRNAs arise from the genomic interval between two genes. Gao et al^13^ described intergenic circRNA as the RNA that contains two intronic circRNA fragments flanked by GT‐AC splicing. Ottesen et al^14^ confirmed four intergenic exons and suggested that the incorporation of each of these intergenic exons could form different intergenic circRNAs. Therefore, intergenic circRNA sequences can be both intronic and exonic.

### 2.3 Characteristics of circRNAs

#### 2.3.1 Evolutionary conservation

(Supplementary file 2).

#### 2.3.2 Structural stability

(Supplementary file 3).

#### 2.3.3 Spatiotemporal specific expression

Though circRNAs show conservation among species, there are also some circRNAs that have species‐, cell‐, tissue‐ and developmental stage‐specific expression. A comparative analysis of the expression of circRNAs from humans and mice found that only a small portion of human circRNAs could be detected in parallel mouse samples, and these circRNAs were conserved. In total, 85% of circRNAs in humans and 60% of circRNAs in mice showed species‐specific expression patterns.^15^ CircRNAs also exhibit cell‐ and tissue‐specific expression patterns. A circRNA catalog of 20 different tissues and cells from a single donor revealed that circRNA expression was highly cell‐ and tissue‐specific.^3^

In addition, circRNAs exhibit dynamic expression patterns during the cardiac differentiation process. Specifically, Lei et al^4^ detected some circRNAs, including circSLC8A1, circCACNA1D, circSPHKAP and circALPK2, that showed cardiac‐specific expression in human induced pluripotent stem cells (hiPSCs). The expression level of these circRNAs increased during cardiac differentiation and as a result, they were highly enriched in hiPSC‐derived cardiomyocytes. A previous study detected differential expression levels of 226 circRNAs during the differentiation of human umbilical cord‐derived mesenchymal stem cells into cardiomyocyte‐like cells.^16^ These findings indicate the potential of certain circRNAs that have significantly different expression levels during the cardiac differentiation process to serve as biomarkers of cardiomyocytes.

Further, the expression of most cardiac circRNAs was triggered by cardiac progenitor differentiation.^17^ Specifically, circRNAs coming from the *TTN* gene were upregulated from the mesoderm stage to the definitive cardiomyocytes.^18^ Thus, it can be inferred that differential expression of certain circRNAs may play a critical role in pathways that are activated during the process of cardiac differentiation.

## 3 MECHANISMS OF CIRCRNAS IN THE OCCURRENCE AND DEVELOPMENT OF CVDS

Some highly expressed circRNAs corresponded to key cardiac genes including *TTN*, *RYR2* and *DMD*, suggesting the essential role of circRNAs in maintaining normal heart function.^18^ Further, differential expression of cardiac circRNAs is strongly related to CVDs. For example, in the heart of an adult mouse, Jakobi et al^19^ reported that the majority of circRNAs could be matched to CVDs‐related host genes including *Ppp2r3a*, *Hectd1* and *RYR2*. Similarly, Maass et al^3^ analysed an assembly of circRNA in the vena cava and right atrium separated from patients with diverse cardiac defects and reported that the altered levels of certain circRNAs contribute to multiple cardiovascular symptoms. For instance, circRNA isoforms derived from the *RYR2* gene were linked to the pathogenesis of atrial fibrillation.^3^ Another study confirmed that 6,234 circRNAs were differentially expressed between a foetal group with congenital ventricular septal defect (VSD) and a normal group.^20^ Abnormal expression profiles of circRNAs were also associated with coronary heart disease and cardiomyocyte hypertrophy.^5^, ^6^ Specially, circSLC8A1, which was found to be the most abundant human cardiac circRNA, abnormally increased in cardiac tissues from individuals with dilated cardiomyopathy.^4^ Collectively, these studies revealed that altered types and quantities of circRNAs are closely correlated with a number of CVDs. Detecting disease‐associated circRNAs may be a potential method to determine pathological status.

Cardiac circRNAs mediate the pathogenesis of CVDs mainly through the following two major mechanisms: circRNA‐miRNA‐mRNA axis and interaction with proteins. CircRNAs can act as a miRNA ‘sponge’, and miRNA captured by circRNAs lose the ability to bind with downstream mRNA. In addition to the circRNA‐miRNA‐mRNA axis, circRNA can also interact with proteins to exert effects on CVDs. (Table 1).

**TABLE 1:** CircRNAs‐involved mechanisms in CVDs

Mechanisms	CircRNAs	Target cells	Diseases	Ref
circRNA‐miRNA‐mRNA axis	hsa‐circ‐0054633‐miR‐218/HO‐1 hsa‐circ‐0054633‐miR‐218/ROBO1	VEC	Atherosclerosis	23
	hsa‐circ‐0010729‐ miR‐186/HIF‐1α			24
	circWDR77‐ miR‐124/FGF‐2	VSMC		26
	circ‐RUSC2‐ miR‐661/SYK			27
	circ‐SATB2‐miR‐939/STIM1	VSMC	CHD	29
	circNCX1‐miR‐133a‐3p/CDIP1	CM	MI	36
	circ‐MFACR‐ miR‐652‐3p/ MTP18			38
	circ‐Nfix‐ miR‐214/Gsk3β			39
	circ‐Hipk3‐ miR‐133a/ CTGF	VEC		40
	circSLC8A1‐miR‐133a/mRNAs	CM	HF	42
	circ‐HRCR‐ miR‐223/mRNAs			43
	hsa‐circ‐0037909‐ miR‐637/mRNAs	VEC	EH	44
	hsa‐circ‐0037911‐miR‐637/mRNAs			45
	circNr1h4‐miR‐155‐5p/Far1	Kidney collecting duct cells	Hypertensive renal injury	46
	circCCDC66‐miR‐342‐3p/CCDC66	VSMC	AAA	50
CircRNA‐protein interaction	circ‐ANRIL/Pes1	^a^	Atherosclerosis	54
	circ‐ACR/Dnmt3B	CM	MI	55
	circ‐Fndc3b/FUS	VEC		56
	circ‐Hipk3/ N1ICD	CM		40
	circ‐Foxp3/ ID‐1,E2F1,FAK,HIF1α	CM	Cardiac senescence	51

Abbreviations: AAA, abdominal aortic aneurysm; CHD, Coronary heart disease; CM, cardiomyocyte; EH, essential hypertension; HF, heart failure; MI, myocardial infarction; VEC, vascular endothelial cell; VSMC, vascular smooth muscle cell.

^a^ The target cell is unclear.

### 3.1 circRNA‐miRNA‐mRNA axis

circRNAs have various miRNA binding sites in their loop structure.^21^ Therefore, circRNAs can bind with miRNAs; through this sponge effect, the cytoplasmic free miRNAs are decreased, resulting in the downstream target mRNAs of miRNAs being upregulated. This chain of interaction is known as a circRNA‐miRNA‐mRNA axis, which exert pivotal effects on diverse CVDs including atherosclerosis, coronary heart disease, myocardial infarction and heart failure.

#### 3.1.1 Atherosclerosis

Vascular endothelial cells and vascular smooth muscle cells (VSMCs) are the two primary cell types in the pathogenesis of atherosclerosis. Multiple circRNA‐miRNA‐mRNA axes effectively regulate the proliferation and migration of VECs and VSMCs, and subsequently change the functional state of blood vessels, modulating the progression of atherosclerosis.

Vascular endothelial cells, which comprise the inner layer of blood vessels, are considered to function as a significant barrier for the vascular wall.^22^ Endothelial dysfunction, including suppression of cell proliferation, cell migration and angiogenesis, serves as an activator of atherosclerosis. VECs are sensitive to hyperglycaemia or hypoxia, and we have demonstrated that these conditions stimulate the change of the expression profile of circRNAs in VECs. A recent study demonstrated that circRNA‐miRNA‐mRNA axis play key roles in high glucose‐induced endothelial injury. Pan et al^23^ reported that the inhibition of the hsa‐circ‐0054633 induced the endothelial dysfunction in the high‐glucose state, while the content of hsa‐circ‐0054633 was increased at a high‐glucose level. These results reflect that the upregulation of hsa‐circ‐0054633 might be an approach for VECs to resist against endothelial injury induced by hyperglycaemic conditions. Further investigations confirmed that increased hsa‐circ‐0054633 bind with cytoplasmic miR‐218, subsequently upregulating the expression of the miR‐218‐target genes: *roundabout 1 (ROBO1)* and *heme oxygenase‐1 (HO‐1)*.^23^ It can be inferred that the upregulation of hsa‐circ‐0054633 reverses the injury of VECs due to high‐glucose conditions by targeting the miR‐218/HO‐1 and miR‐218/ROBO1 axis. Similarly, Dang et al^24^ found that hsa‐circ‐0010729 knockdown efficiently inhibited the proliferation and enhanced apoptosis of hypoxia‐induced human umbilical vein endothelial cells via the miR‐186/HIF‐1α axis. These results all indicate that increased circRNAs activate different circRNA‐miRNA‐mRNA axes to reduce endothelial injury by promoting the proliferation of VECs and reducing apoptosis of VECs, subsequently enhancing the ability of VECs to resist damage.

Vascular smooth muscle cells, which comprise the middle layer of the vascular wall, maintain the tension of blood vessels. The unordered proliferation of VSMCs accelerates the progression of atherosclerosis.^25^ Chen et al^26^ reported nearly 1,000 differentially expressed circRNAs in high glucose‐induced VSMCs. The authors further verified that the knockdown of a markedly upregulated circRNA, circWDR77, could inhibit the proliferation and migration of VSMCs by targeting miR‐124 and fibroblast growth factor 2 (FGF‐2).^26^ Similarly, the high level of circ‐RUSC2 could promote the proliferation and migration of VSMCs via miR‐661/ SYK signals.^27^ Collectively, upregulated circRNAs enhance the disordered proliferation and migration of VSMCs via different circRNA‐miRNA‐mRNA axes to promote the pathological process of atherosclerosis.

#### 3.1.2 Coronary heart disease (CHD)

Compared with the control group, the expression of 171 and 624 circRNAs were markedly decreased and increased in patients with CHD, respectively.^28^ This suggests the potential role of the downregulation or upregulation of circRNAs with the pathological mechanisms underlying CHD. High‐throughput sequencing data suggested the co‐expression patterns of circRNAs and miRNAs in CHD patients.^5^ Mao et al^29^ further verified the interaction between circRNAs and miRNAs in the progression of CHD. The authors found that circ‐SATB2 was downregulated in contractile VSMCs and upregulated in proliferative VSMCs, indicating the pivotal role of circ‐SATB2 in the phenotypic differentiation of VSMCs. Further investigation revealed that circ‐SATB2 acted as an endogenous miR‐939 sponge to sequester miR‐939 and subsequently upregulated the expression of *STIM1*, the target gene of miR‐939.^29^ Multiple studies have confirmed the positive effect of *STIM1* in the migration of proliferative VSMCs.^30^, ^31^, ^32^ The overexpression of circ‐SATB2 significantly inhibited the level of the contractile VSMCs marker, SM22α.^29^ These findings indicate that circ‐SATB2 can promote phenotypic differentiation from contractile to proliferative VSMCs via the circ‐SATB2‐miR‐939‐*STIM1* axis. This phenotypic modulation of VSMCs increases the risk of CHD.^33^

#### 3.1.3 Cardiac ischaemia/reperfusion (I/R) injury and myocardial infarction (MI)

Cardiac ischaemia/reperfusion (I/R) injury acts as a basic pathological mechanism in the occurrence and treatment of MI. Ischaemia leads to the depletion of ATP in myocardial mitochondria, which results in mitochondrial fission. Decreased functional mitochondria then cause the energy shortage of cardiomyocytes and finally induce the apoptosis of cardiomyocytes.^34^ Reperfusion always occurs in the recanalization of the obstructed coronary artery after reperfusion therapy of MI, but it aggravates myocardial damage due to the release of reactive oxygen species (ROS).^35^ Recent research has supported the crucial role of the circRNA‐miRNA‐mRNA axis in I/R injury and MI. Specifically, Li et al^36^ found that circNCX1 was upregulated in correspondence with the increased ROS in I/R injury and promoted the apoptosis of cardiomyocytes through the circNCX1‐miR‐133a‐3p‐*CDIP1* axis. *CDIP1* is known as the pro‐apoptotic gene cell death‐inducing protein, the overexpression of which induces apoptosis. Similarly, differentially expressed circRNAs and miRNAs and their interaction were predicted in acute MI patients.^37^ Wang et al^38^ further identified that an apoptosis‐related circRNA (circ‐MFACR) could enhance the expression of *MTP18* by directly sponging cytoplasmic miR‐652‐3p. Upregulated *MTP18* then induced mitochondrial fission and the apoptosis of cardiomyocytes. These results illustrate the pro‐apoptotic effects of the circRNA‐miRNA‐mRNA axis. Another study verified the downregulation of a super‐enhancer‐regulated conserved circRNA, circ‐Nfix, which could promote the regeneration of cardiomyocytes after MI in adult mice via targeting the miR‐214/Gsk3β axis, indicating a potential therapeutic target for MI.^39^ Besides, Si et al found that circ‐Hipk3 overexpression improved cardiac function after MI and it can serve as miR‐133, a sponge to increase the expression of connective tissue growth factor (CTGF), which is essential for coronary artery endothelial cell function.^40^ Thus, circRNA‐miRNA‐mRNA axis may improve myocardial repair after MI by facilitating angiogenesis.

#### 3.1.4 Heart failure (HF)

MI and pathological hypertrophy promote heart failure (HF). The most abundant human cardiac circRNA, circSLC8A1, is elevated while miR‐133a is downregulated in cardiac hypertrophy.^4^, ^41^ Moreover, upregulated circSLC8A1 sequestered miR‐133a to increase multiple target mRNAs of miR‐133a,^42^ indicating that the circSLC8A1‐miR‐133a‐mRNAs axis serves as a pivotal mechanism in the pathogenesis of cardiac hypertrophy and the inhibition of circSLC8A1 may be a potential method to treat cardiac hypertrophy. In contrast, Wang et al^43^ reported that another circRNA, circ‐HRCR, inhibited cardiac hypertrophy and HF via sequestering endogenous miR‐223. Taken together, different circRNAs may have opposite effects on HF depending on the properties of the protein translated by mRNA in the circRNA‐miRNA‐mRNA axis.

#### 3.1.5 circRNA‐miRNA‐mRNA axis in other CVDs

Recent studies found that hsa‐circ‐0037909 and hsa‐circ‐0037911 were significantly upregulated in patients with essential hypertension compared to healthy group.^44^, ^45^ Subsequent analysis revealed that hsa‐circ‐0037909 and hsa‐circ‐0037911 could contribute to the pathogenesis of essential hypertension via acting as a sponge to inhibit miR‐637 activity.^44^, ^45^ Moreover, Lu et al^46^ found that circNr1h4 was significantly downregulated in the injured kidney of mice with hypertension. CircNr1h4 protected kidney from hypertensive injury via acting as a sponge to inhibit miR‐155‐5p activity and upregulating its target gene fatty acid reductase 1.^46^

Besides, 11 significantly dysregulated circRNAs were identified in septic shock‐induced cardiac depression.^47^ Further analysis found the possible relationship between these circRNAs and their target miRNAs/mRNAs, indicating the important role of circRNA‐miRNA‐mRNA axis in the pathological process of myocardial depression in septic shock.^47^

Moreover, differentially expressed circRNAs were discovered in both mice and human with abdominal aortic aneurysm (AAA).^48^, ^49^ Specially, circCCDC66 was upregulated in AAA.^48^ Further research corroborated that depletion of circCCDC66 reduced apoptosis of VSMC and promoted proliferation of VSMC, indicating a suppressed AAA formation trend.^50^ Besides, circCCDC66 induced the progression of AAA via acting as a miR‐342‐3p sponge to promote the effect of CCDC66 on the proliferation and apoptosis of VSMC.^50^

### 3.2 Interaction between circRNAs and RBPs

CircRNA‐protein interaction is another molecular mechanism relevant to human CVDs. Du et al^51^ found that circ‐Foxp3 was upregulated in cardiac tissues of aged patients, which could interact with the anti‐stress proteins HIF1α and FAK, the anti‐senescent protein ID‐1 and the transcription factor E2F1. These proteins then lost their effect of anti‐stress and anti‐senescent, resulting in cellular senescence. Apart from the role in cardiac senescence, current studies have shown that circRNAs could bind with RBPs to participate in the pathogenesis of CVDs through modulating rRNA maturation, inducing cell autophagy and apoptosis, and mediating angiogenesis and cardiomyocytes regeneration.

#### 3.2.1 Atherosclerosis

The interaction between circRNAs and RBPs could disturb ribosomal biogenesis, thus regulating the pathogenesis of atherosclerosis. The PeBoW complex is essential for ribosomal biogenesis; Pes1 is a nucleolar protein that is a key component of the PeBoW complex.^52^ A previous study has shown that the deletion of the C‐terminal domain of Pes1 effectively inhibited rRNA maturation.^53^ Additionally, it was found that the C‐terminal domain of Pes1 contained binding sites of both pre‐rRNA and circ‐ANRIL.^54^ These results indicate that the interaction between circ‐ANRIL and the C‐terminal domain of Pes1 can modulate the process of turning pre‐rRNA into mature rRNA, subsequently affecting ribosomal biogenesis. Further experiments confirmed that the upregulation of circ‐ANRIL reduced rRNA maturation. The accumulated pre‐rRNA induced nucleolar stress and then stabilized the pro‐apoptotic protein p53.^54^ Consequently, circ‐ANRIL promotes the apoptosis of different cells in the pathological process of atherosclerosis via interacting with Pes1; however, it is unknown whether circ‐ANRIL is atheroprotective in atherosclerosis. For instance, the apoptosis of VECs is pro‐atherosclerotic, whereas the apoptosis of VSMCs is anti‐atherosclerotic.

#### 3.2.2 Myocardial infarction (MI)

Dysregulated autophagy contributes to the progression of CVDs including MI. Zhou et al^55^ reported that an autophagy‐related circular RNA (circ‐ACR) attenuated the autophagy process and the death of cardiomyocytes, resulting in reduced sizes of MIs. Further, Zhou et al^55^ identified the direct binding of circ‐ACR and Dnmt3B. Their interaction blocked the DNA methylation of the Pink1 promoter, which is mediated by Dnmt3B, thereby increasing Pink1 and repressing autophagy via phosphorylating FAM65B.^55^ The circ‐ACR‐Dnmt3B‐Pink1‐FAM65B axis acts as a regulatory factor of autophagy and death of cardiomyocytes, and thus, it participates in the pathological process of MI.

A recent study found a decreased expression level of circ‐Fndc3b in post‐MI heart tissue.^56^ The overexpression of circ‐Fndc3b could increase the level of VEGF, thus promoting angiogenesis and maintaining cardiac function. Detailed mechanisms revealed that the interaction between circ‐Fndc3b and FUS, an RBP that participates in many cellular processes, including angiogenesis and apoptosis, played a crucial role in this process.^56^ Therefore, the circ‐Fndc3b‐FUS‐VEGF axis modulates cardiac repair after MI. Another study found that circ‐Hipk3 could bind with Notch1 intracellular domain (N1ICD) and promoted N1ICD acetylation and nuclear translocation, which is essential for cardiomyocyte proliferation after MI.^40^, ^57^

### 3.3 Translational regulation by circRNAs

CircRNAs can also regulate translation of their linear counterpart through binding with RBPs.^58^, ^59^ Several circRNAs that bind to RBP HuR were verified in HeLa cells.^60^ Among these circRNAs, circPABPN1 was one of the most significantly enriched HuR targets and mechanistically suppressed HuR binding to PABPN1 mRNA to competitively inhibit translation of PABPN1.^60^ Hu et al^61^ found that HuR knockout in cardiomyocytes enhanced isoproterenol‐induced cardiac remodelling. Although several studies have identified that HuR play a role in myocardial remodelling, the role of circRNAs‐HuR complex in CVDs is still unclear.

## 4 CLINICAL PROSPECTS

Compared with linear biomarkers, circRNAs are abundant, conserved and have a stable circular structure, which promotes their use as biomarkers for diagnosis and prognosis of CVDs.

### 4.1 Biomarkers for diagnosis

Coronary computed tomography angiography (CTA), treadmill exercise test (TET), Holter monitoring and electrocardiogram (ECG) are current non‐invasive diagnosis methods for coronary artery diseases (CADs). Sensitivities and specificities of CTA, TET, Holter monitoring and ECG were found to be 0.92 and 0.75,^62^ 0.79 and 0.80,^63^ 0.65 and 0.90^64^ and 0.29 and 0.67,^65^ respectively. Recent studies have reported that several circRNAs detected in peripheral blood could also be used as diagnostic biomarkers for CADs. The sensitivity and specificity of hsa‐circ‐0001879, hsa‐circ‐0004104 and hsa‐circ‐0124644 were 0.83 and 0.54,^28^ 0.71 and 0.61^28^ and 0.87 and 0.77,^66^ respectively. Therefore, these circRNAs have higher diagnostic values than ECG, and similar value as CTA, TET and Holter monitoring. However, using the detection of circRNAs for the diagnosis of CADs does not have high specificity. Therefore, we suggest that it would be most effective to combine the detection of circRNAs with another diagnosis method.

### 4.2 Risk of stratification after myocardial infarction

A considerable number of patients suffer from HF after MI. Recent research has confirmed that a circRNA, circ‐MICRA, was related to the progression of HF after MI and could act as a marker for risk stratification after MI. Antonio et al^67^ classified 472 patients with acute MI into three groups according to their EF value: reduced EF (≤40%), mid‐range EF (41%‐49%) and preserved EF (≥50%). Compared to the reduced EF and preserved EF groups, the circ‐MICRA were relatively lower in the mid‐range EF group, indicating the potential of circ‐MICRA to differentiate patients in the mid‐range EF group from the other two groups; however, additional methods should be applied for the separation of patients in reduced EF and preserved EF group.

## 5 CONCLUSIONS

CircRNAs are a type of non‐coding RNAs with a closed loop structure. Previously published studies have annotated multiple cardiac circRNAs and some circRNAs that show differential expression can be used as biomarkers for the diagnosis of CVDs; however, the specificity of the diagnosis remains a challenge due to the wide distribution of circRNAs in varied cells and tissues. Most studies have revealed the differential expression of certain circRNAs in the plasma or serum, but the origin of these RNAs in circulation has not been clearly clarified. Besides, majority of researches about circRNAs and CVDs just included limited number of patients; thus, meaningful effects of circRNAs in CVDs and the significant value of candidate circRNAs as biomarkers could be ignored. Further studies, especially the large‐scale prospective clinical trials with well‐stratified patients, should be performed to enhance the predictive and diagnostic value of certain circRNAs in CVDs. Moreover, although circRNAs act as miRNA sponge and interact with RNA‐binding proteins, additional pathways remain to be explored in the pathogenesis of CVDs at the molecular level. As for the clinical application, a recent study found that artificial circRNA sponges (circmiRs) which target several miRNAs could extenuate pressure overload‐induced cardiac hypertrophy,^68^ indicating that circmiRs may provide a new therapy for CVDs, with its extended half‐lives and lower requirement of dose compared to current alternatives.

## CONFLICT OF INTEREST

The authors confirm that there are no conflicts of interest.

## AUTHOR CONTRIBUTION

**Ying Tang:** Conceptualization (lead); Data curation (lead); Formal analysis (lead); Funding acquisition (lead); Investigation (lead); Methodology (lead); Project administration (lead); Resources (lead); Software (lead); Supervision (lead); Validation (lead); Visualization (lead); Writing‐original draft (lead); Writing‐review & editing (lead). **Jinghui Bao:** Funding acquisition (supporting). **Jiahui Hu:** Project administration (supporting); Visualization (supporting). **Leiling Liu:** Investigation (supporting); Validation (supporting). **Danyan Xu:** Supervision (equal).

## Supporting information

Supplementary file 1

Supplementary file 2

Supplementary file 3
