# Supplementary material for: Circular RNA in cardiovascular disease: Expression, mechanisms and clinical prospects
Source: J Cell Mol Med. 2020 Dec 22;25(4):1817–24. doi: 10.1111/jcmm.16203 (PMC7882961; doi:10.1111/jcmm.16203)
Supplement: Supplementary file 1 [file JCMM-25-1817-s001.docx]

Supplementary file1

1. ***Intron-pairing-driven circularization***

In intron-pairing-driven circularization, the mechanism is the formation of secondary structures in the pre-mRNA mediated by direct base-pairing of cis-acting elements (short non-coding regulatory sequences) located specifically in the flanking intronic sequence [[1](#_ENREF_1)]. These cis-acting elements can be either short interspersed nuclear elements (SINEs; such as Alu repeats in the human genome) or non-repetitive complementary sequences [[2](#_ENREF_2), [3](#_ENREF_3)]. This process leads to the release of a circular RNA along with a terminated fork by-product rather than a mature mRNA in lariat-driven circularization (Fig.1a).

Tan et al. [[4](#_ENREF_4)] analyzed deep RNA-sequencing data from left ventricular samples of healthy humans and uncovered that 82.32% (12 611/15 318) of the detected circRNAs were derived from exons and the majority of these exonic circRNAs were spliced from coding exons. In other words, these cardiac circRNAs were primarily derived from constitutive exons rather than alternatively spliced exons, indicating that most cardiac circRNAs might be generated at the expense of their linear counterpart. Moreover, the biogenesis process of cardiac circRNAs was mainly flanked by ICSs, indicating that intron-pairing-driven circularization is the major biogenesis model of cardiac circRNAs [[5](#_ENREF_5)].

***b) RBP-driven circularization***

In RBP-driven circularization, some RBPs serve as trans-acting factors that can recognize and bind with cis-acting elements located in the introns flanking the backsplicing exons. Then, the two distant splice sites are brought into close proximity through the interaction of RBPs, followed by steps similar to intron-pairing-driven circularization [[6](#_ENREF_6)]. (Fig.1b) Multiple RBPs, such as RNA-binding protein trinucleotide repeat-containing 6A (TNRC6A) [[6](#_ENREF_6)], RBP Quaking (QKI) [[7](#_ENREF_7)], and Muscleblind protein (MBL) [[8](#_ENREF_8)], facilitate the biogenesis of circRNA through binding with the intronic sequences in the vicinity of circRNA-forming exons. A recent study found that the knockdown of QKI with siRNA or the mutation of the binding sites of QKI efficiently inhibited the expression of cardiac circRNAs, further illustrating the role of RBP-driven circularization in cardiac circRNA biogenesis [[9](#_ENREF_9)].

***c) Lariat-driven circularization***

In lariat-driven circularization, research has found that circRNAs, known as circular intronic RNAs (ciRNAs), originate directly from the removed intron when there are no exon-skipping events in pre-mRNA splicing [[10](#_ENREF_10)]. (Fig.1c) CircRNAs are also generated from the exon-containing lariats that originate during exon-skipping events [[11](#_ENREF_11)]. Some introns of the exon-containing lariat are removed, forming an exonic or exon-intron circRNA [[12](#_ENREF_12)]. (Fig.1d)

Proteins can modulate this exon-skipping process. For instance, more than 400 diverse exonic circRNAs were generated from the transcription of the human *Titin* (*TTN*) gene in cardiomyocytes. In this process, RNA-Binding Motif protein 20 (RBM20) served as the most significant splicing element for the circularization of circRNAs [[4](#_ENREF_4), [13](#_ENREF_13)]. Further investigations found that in RBM20 knockout mice, the expression of a special category of circRNAs from the *TTN* transcript were lost; all of the lost circRNAs were derived from the PEVK domain and Ig repeats within the I-band region of the *TTN* transcript, a region which is responsible for alternative splicing [[14](#_ENREF_14), [15](#_ENREF_15)]. Thus, it can be inferred that unlike the majority of cardiac circRNAs arising from constitutive exons, the production of circRNAs from the *TTN* gene correspond to the skipped exons in the alternative splicing process [[5](#_ENREF_5)]. Moreover, Aufiero et al. [[5](#_ENREF_5)] found that the circularization of cardiac circRNAs from other genes, such as *Stk39*, *Fan1*, *Sorbs1*, and *Xdh*, were also the target of RBM20. Together, these results demonstrated the key roles of RBM20 in the exon-skipping model of the formation of cardiac circRNAs.

Multiple regulatory factors modulate the expression of circRNAs both in cardiomyocytes and human vascular endothelial cells (hVECs), two major cells involved in CVDs. RBM20 regulates alternative splicing to participate in the circularization of circRNAs in cardiomyocytes, while hypoxia and high-glucose conditions may induce changes in the expression profiles of circRNAs in hVECs.

In hVECs, hypoxia is a crucial stimulation for the expression of circRNAs. To better understand the effect of hypoxia on the biogenesis of endothelial circRNAs, scientists exposed human umbilical vein endothelial cells to a hypoxic environment and found that the expression level of circRNAs significantly changed [[16](#_ENREF_16), [17](#_ENREF_17)]. Multiple studies have confirmed that hypoxia could modulate the splicing of pre-mRNA [[18](#_ENREF_18)]. Hypoxia regulates splicing factors, such as serine arginine (SR) proteins [[19](#_ENREF_19)]. In addition, hypoxia influences the activity of molecules that bind to splicing factors such as the Jumonji domain-containing protein 6 (Jmjd6) [[20](#_ENREF_20)]. Thus, it can be inferred that hypoxia might exert effects on the backsplicing process and then the circularization of circRNAs, but the mechanism requires further exploration. Moreover, Shang et al. [[21](#_ENREF_21)] compared the expression catalogs of circRNAs of a high glucose-induced group and a control group in hVECs and found dramatically different expression profiles of circRNAs, indicating that high glucose is another factor that alters the level of circRNAs in human endothelial cells.

**References**

1. Jeck WR, Sorrentino JA, Wang K, Slevin MK, Burd CE, Liu J, Marzluff WF, Sharpless NE: **Circular RNAs are abundant, conserved, and associated with ALU repeats**. *RNA (New York, NY)* 2013, **19**(2):141-157.

2. Ivanov A, Memczak S, Wyler E, Torti F, Porath HT, Orejuela MR, Piechotta M, Levanon EY, Landthaler M, Dieterich C *et al*: **Analysis of intron sequences reveals hallmarks of circular RNA biogenesis in animals**. *Cell reports* 2015, **10**(2):170-177.

3. Zhang XO, Wang HB, Zhang Y, Lu X, Chen LL, Yang L: **Complementary sequence-mediated exon circularization**. *Cell* 2014, **159**(1):134-147.

4. Tan WL, Lim BT, Anene-Nzelu CG, Ackers-Johnson M, Dashi A, See K, Tiang Z, Lee DP, Chua WW, Luu TD *et al*: **A landscape of circular RNA expression in the human heart**. *Cardiovascular research* 2017, **113**(3):298-309.

5. Aufiero S, van den Hoogenhof MMG, Reckman YJ, Beqqali A, van der Made I, Kluin J, Khan MAF, Pinto YM, Creemers EE: **Cardiac circRNAs arise mainly from constitutive exons rather than alternatively spliced exons**. *RNA (New York, NY)* 2018, **24**(6):815-827.

6. Dai X, Zhang N, Cheng Y, Yang T, Chen Y, Liu Z, Wang Z, Yang C, Jiang Y: **RNA-binding Protein Trinucleotide repeat-containing 6A Regulates the Formation of Circular RNA 0006916, with Important Functions in Lung Cancer Cells**. *Carcinogenesis* 2018.

7. Conn SJ, Pillman KA, Toubia J, Conn VM, Salmanidis M, Phillips CA, Roslan S, Schreiber AW, Gregory PA, Goodall GJ: **The RNA binding protein quaking regulates formation of circRNAs**. *Cell* 2015, **160**(6):1125-1134.

8. Ashwal-Fluss R, Meyer M, Pamudurti NR, Ivanov A, Bartok O, Hanan M, Evantal N, Memczak S, Rajewsky N, Kadener S: **circRNA biogenesis competes with pre-mRNA splicing**. *Molecular cell* 2014, **56**(1):55-66.

9. Gupta SK, Garg A, Bar C, Chatterjee S, Foinquinos A, Milting H, Streckfuss-Bomeke K, Fiedler J, Thum T: **Quaking Inhibits Doxorubicin-Mediated Cardiotoxicity Through Regulation of Cardiac Circular RNA Expression**. *Circulation research* 2018, **122**(2):246-254.

10. Zhang Y, Zhang XO, Chen T, Xiang JF, Yin QF, Xing YH, Zhu S, Yang L, Chen LL: **Circular intronic long noncoding RNAs**. *Molecular cell* 2013, **51**(6):792-806.

11. Zaphiropoulos PG: **Circular RNAs from transcripts of the rat cytochrome P450 2C24 gene: correlation with exon skipping**. *Proceedings of the National Academy of Sciences of the United States of America* 1996, **93**(13):6536-6541.

12. Barrett SP, Wang PL, Salzman J: **Circular RNA biogenesis can proceed through an exon-containing lariat precursor**. *eLife* 2015, **4**:e07540.

13. Werfel S, Nothjunge S, Schwarzmayr T, Strom TM, Meitinger T, Engelhardt S: **Characterization of circular RNAs in human, mouse and rat hearts**. *Journal of molecular and cellular cardiology* 2016, **98**:103-107.

14. LeWinter MM, Granzier HL: **Titin is a major human disease gene**. *Circulation* 2013, **127**(8):938-944.

15. Khan MA, Reckman YJ, Aufiero S, van den Hoogenhof MM, van der Made I, Beqqali A, Koolbergen DR, Rasmussen TB, van der Velden J, Creemers EE *et al*: **RBM20 Regulates Circular RNA Production From the Titin Gene**. *Circulation research* 2016, **119**(9):996-1003.

16. Boeckel JN, Jae N, Heumuller AW, Chen W, Boon RA, Stellos K, Zeiher AM, John D, Uchida S, Dimmeler S: **Identification and Characterization of Hypoxia-Regulated Endothelial Circular RNA**. *Circulation research* 2015, **117**(10):884-890.

17. Dang RY, Liu FL, Li Y: **Circular RNA hsa_circ_0010729 regulates vascular endothelial cell proliferation and apoptosis by targeting the miR-186/HIF-1alpha axis**. *Biochemical and biophysical research communications* 2017, **490**(2):104-110.

18. Memon D, Dawson K, Smowton CS, Xing W, Dive C, Miller CJ: **Hypoxia-driven splicing into noncoding isoforms regulates the DNA damage response**. *NPJ genomic medicine* 2016, **1**:16020.

19. Jakubauskiene E, Vilys L, Makino Y, Poellinger L, Kanopka A: **Increased Serine-Arginine (SR) Protein Phosphorylation Changes Pre-mRNA Splicing in Hypoxia**. *The Journal of biological chemistry* 2015, **290**(29):18079-18089.

20. Boeckel JN, Guarani V, Koyanagi M, Roexe T, Lengeling A, Schermuly RT, Gellert P, Braun T, Zeiher A, Dimmeler S: **Jumonji domain-containing protein 6 (Jmjd6) is required for angiogenic sprouting and regulates splicing of VEGF-receptor 1**. *Proceedings of the National Academy of Sciences of the United States of America* 2011, **108**(8):3276-3281.

21. Shang FF, Luo S, Liang X, Xia Y: **Alterations of circular RNAs in hyperglycemic human endothelial cells**. *Biochemical and biophysical research communications* 2018, **499**(3):551-555.

22. Aufiero S, Reckman YJ, Pinto YM, Creemers EE: **Circular RNAs open a new chapter in cardiovascular biology**. 2019, **16**(8):503-514.


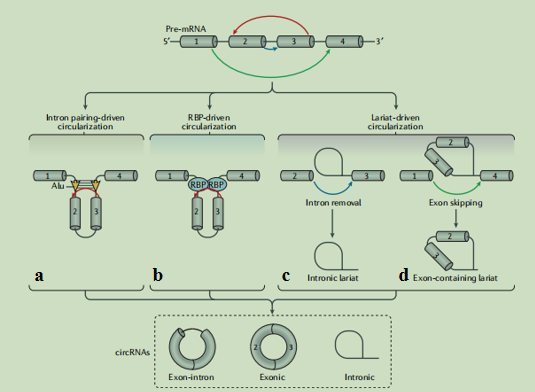


**Figure Legend**

Fig. 1 The biogenesis of circRNAs [[22](#_ENREF_22)].

a. In intron-pairing-driven circularization, the base pair of cis-acting elements occurs first. Then, the 2ʹ OH of the upstream intron reacts with the 5ʹ phosphate of the downstream intron, followed by the 3ʹ OH of the 3ʹ-exon reacting with the 5ʹ phosphate of the 5ʹ-exon. Then, a circular RNA and a terminated fork by-product are released. Finally, with the removal of some introns in the circular RNA, an exonic or exon-intron circRNA is produced.

b. In RBP-driven circularization, the intronic sequences recruit RNA binding proteins (RBPs). Then, the upstream and downstream RBP-intron complex bind with each other. The remainder of the process follows the same steps as intron-pairing-driven circularization.

c. In lariat-driven circularization, an intronic RNA lariat circularized with 2ʹ,5ʹ-phosphodiester is released and subsequently the 3ʹ-tail of the RNA is removed from the lariat, forming circular intronic RNAs (ciRNAs).

d. In lariat-driven circularization, the first step is the canonical splicing of pre-mRNA. Second, in the RNA lariat, the 2ʹ OH of the 5ʹ-intron attacks the 5ʹ phosphate of the 3ʹ-intron, followed by the 5ʹ phosphate of the 5ʹ-exon reacting with the 3ʹ OH of the 3ʹ-exon. As a result, an exon-containing RNA is produced with the release of an intronic double lariat by-product. Finally, some introns of the circular RNA are removed, forming an exonic or exon-intron circRNA.
