# Supplementary material for: Circular RNA in cardiovascular disease: Expression, mechanisms and clinical prospects
Source: J Cell Mol Med. 2020 Dec 22;25(4):1817–24. doi: 10.1111/jcmm.16203 (PMC7882961; doi:10.1111/jcmm.16203)
Supplement: Supplementary file 2 [file JCMM-25-1817-s002.docx]

Supplementary file 2

With the development of high-throughput sequencing methods, numerous circRNAs have been discovered to be abundant in nematodes [[1](#_ENREF_1)], mammals [[1](#_ENREF_1), [2](#_ENREF_2)], Drosophila [[2](#_ENREF_2)] and plants [[3](#_ENREF_3)]. A sequence analysis comparing homologous sequences of circRNAs among different species has indicated that circRNAs are conserved. A recent study compared circRNAs between humans and mice, and found that 15% of circRNAs were conserved in humans and 40% of circRNAs were conserved in mice [[4](#_ENREF_4)]. Similarly, by comparing the back splicing site of circRNAs, Liu et al. [[5](#_ENREF_5)] found that 12,348 of 140,790 human circRNAs were conserved between humans and mice. The conserved nature of circRNAs indicates that circRNAs may participate in the maintenance of basic biological functions shared by different species; however, further research is needed on this topic.

**References**

1. Memczak S, Jens M, Elefsinioti A, Torti F, Krueger J, Rybak A, Maier L, Mackowiak SD, Gregersen LH, Munschauer M *et al*: **Circular RNAs are a large class of animal RNAs with regulatory potency**. *Nature* 2013, **495**(7441):333-338.

2. Rybak-Wolf A, Stottmeister C, Glazar P, Jens M, Pino N, Giusti S, Hanan M, Behm M, Bartok O, Ashwal-Fluss R *et al*: **Circular RNAs in the Mammalian Brain Are Highly Abundant, Conserved, and Dynamically Expressed**. *Molecular cell* 2015, **58**(5):870-885.

3. Zhang X, Ma X, Ning L, Li Z, Zhao K, Li K, He J, Yin D: **Genome-wide identification of circular RNAs in peanut (Arachis hypogaea L.)**. *BMC genomics* 2019, **20**(1):653.

4. Dong R, Ma XK: **Increased complexity of circRNA expression during species evolution**. *RNA biology* 2017, **14**(8):1064-1074.

5. Liu M, Wang Q, Shen J, Yang BB, Ding X: **Circbank: a comprehensive database for circRNA with standard nomenclature**. *RNA biology* 2019, **16**(7):899-905.
