# Supplementary material for: Circular RNA in cardiovascular disease: Expression, mechanisms and clinical prospects
Source: J Cell Mol Med. 2020 Dec 22;25(4):1817–24. doi: 10.1111/jcmm.16203 (PMC7882961; doi:10.1111/jcmm.16203)
Supplement: Supplementary file 3 [file JCMM-25-1817-s003.docx]

Supplementary file 3

Most circRNAs cannot be degraded by RNase R and even the poly(A) + RNA Depletion treatment, which can almost entirely digest linear RNA, illustrating the stable structure of circRNAs [[1](#_ENREF_1), [2](#_ENREF_2)]. Compared with linear RNAs, the covalently closed 5ʹend-to-3ʹend circular structure of circRNAs serves as the basis of their structural stability. Therefore, this characteristic may place circRNAs at the forefront of potential biomarkers for diagnosis of diseases including CVDs. A recent study even confirmed that engineering exogenous circRNA could be expressed as a much more robust and stable protein than linear RNAs in eukaryotic cells, indicating the possibility of circRNAs for the treatment of CVDs [[3](#_ENREF_3)].

**References**

1. Zhang Y, Zhang XO, Chen T, Xiang JF, Yin QF, Xing YH, Zhu S, Yang L, Chen LL: **Circular intronic long noncoding RNAs**. *Molecular cell* 2013, **51**(6):792-806.

2. Panda AC, De S, Grammatikakis I, Munk R, Yang X, Piao Y, Dudekula DB, Abdelmohsen K, Gorospe M: **High-purity circular RNA isolation method (RPAD) reveals vast collection of intronic circRNAs**. *Nucleic acids research* 2017, **45**(12):e116.

3. Wesselhoeft RA, Kowalski PS, Anderson DG: **Engineering circular RNA for potent and stable translation in eukaryotic cells**. *Nature communications* 2018, **9**(1):2629.
